# Medical knowledge on managing anaphylaxis in northeastern brazil

**DOI:** 10.1186/1939-4551-8-S1-A249

**Published:** 2015-04-08

**Authors:** Maria Do Socorro Viana Silva De Sá, Antônia Correia Da Silva

**Affiliations:** 1 Faculdade de Ciências Médicas de Campina Grande, Campina Grande, Paraiba, 58400-250, Brazil; 2 Faculdade de Ciências Médicas de Campina Grande, Maceió, Alagoas, 57055-667,Brazil

## Background

To assess the medical knowledge on the management of anaphylaxis in emergency (non-pharmacological actions, pharmacological approaches, biphasic anaphylaxis reaction) at the Hospital de Trauma e Emergência Dom Gonzaga Fernandes, located in Campina Grande-PB.

## Methods

A descriptive cross-sectional study with a questionnaire to 60 physicians working in Hospital de Trauma e Emergência Dom Gonzaga Fernandes em Campina Grande-PB.

## Results

No pharmacological actions, pharmacological measures, and knowledge of biphasic anaphylaxis reaction were approached. As non-pharmacological measures, maintenance of respiratory tract was cited by 48 physicians, whereas attention to hydration, intravenous access and patient positioning were cited by 12 doctors. As pharmacological measure, adrenaline was mentioned in 48 questionnaires as the primary drug, nine physicians reported corticosteroids as primary drug and three physicians cited antihistamine. As a route of epinephrine administration, they were mentioned: subcutaneous (39,6%), intravenous (22,9%), intramuscular (29,2%) and 8,3% did not know. About the local administration of epinephrine: 70,8% did not answer, 10,4% cited the forearm, 6,3% cited the abdomen and 12,5% cited the thigh. About patients with beta-blockers and refractory to treat anaphylaxis, an indication of glucagon to control framework was cited by four doctors. Approximately 83% of respondents were unaware of the biphasic reaction of anaphylaxis and 16% mentioned that meet only 12% described it correctly.

## Conclusions

Anaphylaxis is a potentially fatal entity, whose incidence and severity are increasing, but remains significantly under-diagnosed and under-treated.

